# Surgical site infections occurrence and associated risk factors: a matched case-control study

**DOI:** 10.1017/ash.2025.10269

**Published:** 2026-01-12

**Authors:** Nassab Fakhreddine, Hani Dimassi, Wissam Kabbara, Rola Husni, Sanaa Zoghbi, Hanine Mansour

**Affiliations:** 1 Lebanese American University School of Pharmacy, Byblos, Lebanon; 2 Lebanese American University Medical Center – Rizk Hospital, Beirut, Lebanon

## Abstract

**Objective:**

Surgical site infections (SSIs) pose a significant healthcare challenge, raising patient morbidity, mortality, and costs. Various intrinsic, patient-specific, and perioperative factors contribute to SSIs. This study aims to identify SSI-associated risk factors, microorganism types, and antibiotic susceptibility patterns in surgical patients.

**Design:**

This is a matched-case control study.

**Setting:**

The Lebanese American University Medical Center – Rizk Hospital, Beirut Lebanon.

**Patients:**

The study included surgical patients.

**Methods:**

This retrospective case-control study analyzed data from surgical patients over a five-year period, matching 113 SSI cases with controls in a 1:3 ratio by gender and surgery type.

**Results:**

Among 324 patients (81 cases vs 243 controls), hypoalbuminemia (<3.5 g/dL) and age ≥65 years were significantly associated with SSIs (*P* = .025 and *P* = .039), respectively. Antibiotic redosing was associated with lower odds of SSIs (OR = 0.19, *P* = .042), indicating a potential protective effect.

**Conclusions:**

Our findings were consistent with similar studies. Elderly patients and those with hypoalbuminemia were found to be at significantly higher risk of SSIs. Also, antibiotic redosing during prolonged surgeries was associated with reduced SSI risk. In terms of SSI rates, gastrointestinal surgeries (GIs) were the highest with 42.3% of GI cases followed appropriate antibiotic protocols. Like other studies, predominant microorganisms at wound site included *E. coli* and *coagulase-negative staphylococci*.

## Introduction

Surgical site infections (SSIs) occur at or near the surgical incision within 30 days postsurgery—or up to a year if prosthetic materials are involved—impacting the incision site and deeper tissues, organs, and spaces.^1^ Recognized by the World Health Organization (WHO) as a significant global health issue, SSIs dramatically worsen patient outcomes: individuals who develop an SSI are twice as likely to die, 60% more likely to require intensive care, and five times more likely to be readmitted to the hospital than patients without an SSI.^2–4^ In the United States alone, SSIs are among the leading causes of hospital-acquired infections, with over 110,000 cases reported in 2015, adding between $3.5 billion and $10 billion to annual healthcare costs.^5,6^ The burden is particularly severe in developing countries like Africa, where SSIs affect 7.2% of surgical patients.^7^

SSIs can be prevented by following a pre-, intra-, and postoperative surgical site infection prevention bundle which includes but is not limited to using an appropriate surgical antibiotic prophylaxis within an appropriate time frame, cleansing of the surgical site with antiseptics, and maintaining appropriate care of incision postsurgery.^8,9^ Research found that implementing these evidence-based strategies may prevent 55% of SSIs.^6^

Guidelines recommend that preoperative antibiotics be administered in the 60-minute window immediately prior to incision. For longer surgeries, antibiotic redosing is required particularly when the surgery exceeds two half-lives of the agent.^10,11^ Furthermore, prolongation of antibiotic prophylaxis after surgery is not recommended as it increases the risk of resistance and side effects.^1,12^ Antibiotic prophylaxis may be extended for up to 24 hours postsurgery or up to 48 hours in specific cases, such as cardiothoracic procedures.^10^

Several factors contribute to the risk of SSIs, which can be categorized into intrinsic, patient-specific (advanced age, obesity, diabetes, immunosuppressive therapy, hypoalbuminemia, smoking, and massive blood transfusion), and perioperative factors associated with surgical practices (inadequacy in surgical scrubbing, antiseptic skin preparation, antimicrobial prophylaxis, and the duration of the surgical procedure).^13^ Studies show that approximately 7%–15% of patients who undergo major surgical procedures are likely to encounter a postoperative complication, even when aseptic techniques and suitable antimicrobial prophylaxis are employed with a reported postoperative mortality rate ranging from 0.79% to 5.7%.^14,15^

Our medical center has implemented a surgical site infection prevention bundle, along with comprehensive protocols and policies for antibiotic prophylaxis in surgical procedures. First, the surgical site infection prevention bundle checklist is categorized into a preop bundle (patient showering, hair removal, IV antibiotic prophylaxis within 60 mins, and handwashing), an intra-op bundle (traffic flow, closed doors, appropriate room temperature, skin disinfecting before incision, adequate patient’s temperature and blood sugar, and antibiotic redosing), and a postop bundle (handwashing, protection of incision, and discontinuation of antibiotics within 24 h). Second, antibiotic prophylaxis protocols for surgical interventions focus on organism coverage, local epidemiology, antibiotics of choice for both adults and pediatrics, the time window for preoperative antibiotic administration, and recommendations for their use postsurgery. Moreover, the protocols are categorized based on the type of surgery (eg, cardiac, orthopedic, urology) and the assessment of risk factors for infection/colonization with ESBL and MRSA producing organisms for both pediatric and adult patient population.

Given these findings surrounding surgical wound infections, our study aims to evaluate the risk factors associated with the occurrence of surgical site infections in patients who underwent surgery over the last five years at our hospital.

## Methodology

### Study design and setting

This retrospective single-center matched case-control trial was conducted at a 100-bed teaching hospital in Beirut, Lebanon, from October 2023 to February 2024. Data from 20,000 surgical patients were collected using paper-based medical records between 2018 and 2023 and were analyzed, with 113 patients developing SSIs. As per the National Healthcare Safety Network (NHSN) criteria for defining SSI, infections are classified as superficial (skin/subcutaneous tissue, purulence or positive culture, plus localized pain/tenderness) or deep (deep soft tissue infection within 30–90 d, with at least one of the following: abscess, purulence, organism(s) identified from the deep site with signs or symptoms such as fever, tenderness or localized pain, or other evidence including histology or radiation).^16^ Patients were matched in a 1:3 ratio based on gender and type of surgery. Data on patient demographics, surgical site infection risk factors, type of surgery, antibiotics characteristics, and microorganisms detected, and their susceptibility patterns were collected from medical records, the operation room, and infection control databases at our medical center. Data collection was performed by two authors: a pharmacist and an infection control personnel member, who independently extracted data from the medical records, and operation room (OR) reports. The study protocol was approved by the Institutional Review Board of our institution.

### Patient population

Patients aged 18 years and above who underwent surgery at our medical center since 2018 and received antibiotics for surgical infection prevention were included. Patients undergoing ophthalmologic surgery, biopsies, or presenting with infected wounds prior to surgery, and/or currently receiving antibiotics were excluded from this study.

### Endpoints

The primary endpoint was to identify risk factors associated with the occurrence of SSIs, including diabetes, immunosuppression, obesity, smoking, intraoperative bleeding events necessitating blood transfusion, prolonged surgical duration (>2 h), elderly age (≥65 yr), hypoalbuminemia (<3.5 g/dl), appropriate antibiotic use and redosing during surgery, compliance with the hospital’s SSI prevention bundle, and the type of surgery with the highest number of SSIs. The secondary endpoint was to characterize the different types of microorganisms causing SSIs and their susceptibility patterns. Compliance with the hospital’s SSI prevention bundle was assessed using a standardized intraoperative checklist routinely completed by OR nursing staff for every surgical case. Only variables that were consistently documented for all surgical patients were included in the analysis. Several perioperative factors—such as OR traffic, hand hygiene, continuous temperature logs, and perioperative glucose control—were not recorded at the individual patient level and were therefore unavailable for extraction. To avoid bias, no imputation was performed, and analyses were limited to variables with complete documentation. The absence of these process-level factors is acknowledged as a study limitation.

### Statistical analysis

Data analysis was performed using Statistical Package for Social Sciences (SPSS, version 28). Descriptive analysis was performed to explore sample characteristics with means and standard deviations calculated for numerical variables, and frequencies and percentages for categorical data. The bivariate analysis included paired T-test and McNemar Chi-Square test. The multivariable analysis utilized conditional logistic regression. All data were analyzed on the 0.05 significance level.

## Results

### Sample size and enrollment

**Figure 1.**
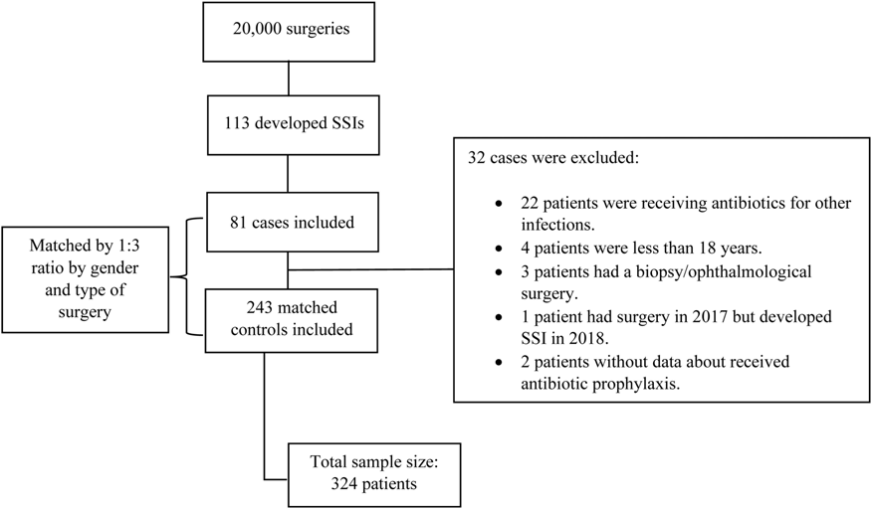
Flowchart of study identification, matching, inclusion, and exclusion criteria.

A total of 324 patients were enrolled in the study. Among them, 113 patients developed SSI. Of these, 32 patients were excluded based on predetermined exclusion criteria, resulting in the inclusion of 81 cases. The cases were matched in a 1:3 ratio to 243 controls. Figure 1. represents the study recruitment flowchart.

### Baseline characteristics

There were no significant differences in smoking status (*P* = .820), antibiotic allergies (*P* = .200), specific types of antibiotic allergies (*P* = .533), or mortality (*P* = .800) between cases and controls. The mean age was 63.56 years (Standard Deviation (SD) = 14.58) for cases and 61.29 years (SD = 15.01) for controls. The mean BMI was 27.84 (SD = 5.45) for cases and 27.83 (SD = 6.15) for controls (Table 1).

**Table 1.** Baseline characteristics for patients’ cases and control groups

Variables	Cases	Controls	*P*-value
	N (%)	N (%)	
**Smoker**			
Yes	34 (43.6)	96 (42.1)	0.820
No	44 (56.4)	132 (57.9)	
**Antibiotic allergy**			
Yes	11 (13.6)	21 (8.7)	0.200
No	70 (86.4)	221 (91.3)	
**Type of allergy**			
Amoxicillin/clavulanate	6 (7.4)	9 (3.7)	
Cefuroxime	1 (1.2)	1 (0.4)	
Ciprofloxacin	0 (0.0)	2 (0.8)	
Moxifloxacin	0 (0.0)	1 (0.4)	
Penicillin	2 (2.5)	5 (2)	
**Death**	1 (1.2)	4 (1.6)	0.800
	Mean (SD^‡^)	Mean (SD^‡^)	
**Age**	63.56 (14.58)	61.29 (15.01)	0.240
**BMI**	27.84 (5.45)	27.83 (6.15)	0.999

^‡^SD, Standard deviation.

### Primary endpoints

#### Risk factors

There was no significant difference in the prevalence of diabetes, immunosuppression, obesity, smoking, bleeding events during surgery, and surgeries lasting more than two hours between cases with SSIs and controls. However, there was a trend toward a statistical significance between age ≥65 years and SSIs (*P* = .06). Moreover, hypoalbuminemia presented a statistically significant risk factor for SSIs (*P* = .025), with unadjusted OR for the association between hypoalbuminemia and SSIs of 3.76 (95% CI: 1.14–12.42).

#### Surgical site infection prevention bundle checklist

Patient showering one day before surgery, immediate removal of hair before the operation, and disinfection of the skin before incision, did not show a significant association with the occurrence of SSIs. The other components of the surgical site infection prevention bundle checklist [Table tbl2]could not be assessed due to the absence of available data (Table 2).

**Table 2. tbl2:** Risk factors and the surgical site infection prevention bundle checklist for cases and control groups

Variables	Cases	Controls	*P*-value	Unadjusted OR	95% CI
	N (%)	N (%)			
**Diabetes**	26 (32.1)	65 (26.7)	0.354	1.29	(0.75–2.23)
**Immunosuppressed**	15 (18.5)	31 (12.8)	0.198	1.55	(0.79–3.05)
**Obesity (BMI > 30 Kg/m** ^2^ **)**	22 (33.8)	59 (28.1)	0.374	1.30	(0.72–2.37)
**Smoking**	34 (43.6)	96 (42.1)	0.819	1.06	(0.63–1.78)
**Bleeding events during surgery**	8 (12.3)	18 (8.8)	0.401	1.46	(0.60–3.53)
**Surgery lasting more than two hours**	72 (88.9)	204 (84)	0.279	1.52	(0.70–3.31)
**Elderly (≥65 years)**	45 (55.6)	106 (43.6)	0.062	1.61	(0.97–2.68)
**Hypoalbuminemia (<3.5 g/dL)**	20 (80)	17 (51.5)	0.025	3.76	(1.14–12.42)
**Patient showering one day before surgery (with chlorhexidine 4% for orthopedic, general surgery, neurosurgery and open-heart surgeries)**			0.631	1.51	(0.27–8.41)
Yes	79 (97.5)	239 (98.4)			
Not available	2 (2.5)	4 (1.6)			
**Removal of hair immediately before the operation with electrical clipper (If hair is removed)**			0.419	*	
No	0 (0.0)	1 (0.4)			
Yes	75 (92.6)	232 (95.5)			
Not available	6 (7.4)	10 (4.1)			
**Surgeon hand wash or hand-rub hands based on the WHO 5 moments: preop and postop**			--	*	
No	0 (0.0)	0 (0.0)			
Yes	0 (0.0)	0 (0.0)			
Not available	81 (100)	243 (100)			
**Control of traffic flow: Do not exceed 11 persons**			--	*	
No	0 (0.0)	0 (0.0)			
Yes	0 (0.)	0 (0.0)			
Not available	81 (100)	243 (100)			
**Maintain closed doors except for necessary movement of staff and supplies**			--	*	
No	0 (0.0)	0 (0.0)			
Yes	0 (0.0)	0 (0.0)			
Not available	81 (100)	243 (100)			
**Maintain temperature 20°C to 23°C, humidity 30% to 60%**			--	*	
No	0 (0.0)	0 (0.0)			
Yes	0 (0.0)	0 (0.0)			
Not available	81 (100)	243 (100)			
**Use antimicrobial scrub or alcohol-based hand rub for surgical hand antisepsis before donning sterile gloves**			--	*	
No	0 (0.0)	0 (0.0)			
Yes	0 (0.0)	0 (0.0)			
Not available	81 (100)	243 (100)			
**Disinfect skin immediately prior to incision with Chlorhexidine or Povidone Iodine (only on mucosa)**			0.430	*	
No	0 (0.0)	1 (0.4)			
Yes	79 (97.5)	240 (98.8)			
Not available	2 (2.5)	2 (0.8)			
**Maintain patient’s body temperature at 36°C**			0.070	*	
No	0 (0.0)	0 (0.0)			
Yes	78 (96.3)	241 (99.2)			
Not available	3 (3.7)	2 (0.8)			
**Ensure the patient’s blood glucose level in normal throughout the operation (diabetic patients only)**			--	*	
No	0 (0.0)	0 (0.0)			
Yes	0 (0.0)	0 (0.0)			
Not available	81 (100)	243 (100)			
**Protect primary closure incision with sterile dressing for 24–48 hrs postop**			--	*	
No	0 (0.0)	0 (0.0)			
Yes	0 (0.0)	0 (0.0)			
Not available	81 (100)	243 (100)			

OR, Odds Ratio. *OR could not be estimated.

#### Operative characteristics

Cases were less likely to receive correct antibiotics and doses than controls, but the difference was not statistically significant (*P* = .148). Antibiotic redosing also differed between the two groups, with only 3.7% of cases receiving appropriate redosing compared to 11.5% of controls; however, this difference was not statistically significant (*P* = .291). Cases were more likely to have antibiotics continued for more than 24 hours postoperatively with an indication to continue compared to controls (*P* = .008) (Table 3).

**Table 3.** Results of the operative characteristics

Variables	Cases	Controls	*P*-value	Unadjusted OR	95% CI
	N (%)	N (%)			
**Correct antibiotics of choice**	46 (56.8)	156 (64.2)	0.233	0.73	(0.44–1.22)
**Correct dose of antibiotics**	79 (97.5)	241 (99.2)	0.245	0.33	(0.04–2.36)
**Correct antibiotics and dose of choice**	44 (54.3)	154 (63.4)	0.148	0.69	(0.41–1.14)
**Antibiotics timing**					
Within 30 minutes	21 (25.9)	76 (31.4)	0.651	1.30	(0.73–2.3)
More than 30–60 minutes	57 (70.4)	158 (65.3)			
Not available	3 (3.7)	8 (3.3)			
**Antibiotic redosing**					
No, no need	45 (83.3)	138 (75.8)	0.291		
No, but should have been done	7 (13.0)	23 (12.6)			
Yes, done	2 (3.7)	21 (11.5)			
**Antibiotics discontinuation**					
No, but there is an indication to continue	6 (7.4)	3 (1.2)	0.008		
No, and no indication to continue	47 (58.0)	132 (54.5)			
Yes	28 (34.6)	107 (44.2)			

A multivariate logistic regression analysis was conducted to assess the adjusted ORs and 95% CIs for variables associated with the occurrence of SSIs (Table 4). The following results were obtained:

Elderly patients (≥65 yr) and those with hypoalbuminemia (<3.5 g/dL) had significantly higher adjusted odds of developing SSIs with an adjusted OR of 2.0 (95% CI: 1.03–3.86, *P* = .039) and 5.3 (95% CI: 1.10–25.08, *P* = .037), respectively.

**Table 4.** Multivariate analysis assessing *the variables associated with the occurrence of SSIs*

Variables	Adjusted OR	95% CI	*P*-value
Elderly (≥65 yrs)	2.0	(1.03–3.86)	0.039
Hypoalbuminemia (<3.5 g/dL)	5.3	(1.10–25.08)	0.037
Antibiotic redosing			
No need (ref)	1.00	--	
No, but should have been done	0.71	(0.27–1.89)	0.496
Yes, done	0.19	(0.04–0.94)	0.042

After controlling for other variables, the need for antibiotic redosing emerged as a significant risk factor for SSIs. Patients who required redosing of antibiotics had significantly lower adjusted odds of developing SSIs compared to those for whom redosing was not needed (OR = 0.19, 95% CI: 0.04–0.94, *P* = .042).

To evaluate which type of surgery had the highest number of SSIs, we compared the total observed number and observed percentage of infections to the predictable percentage based on the distribution of surgeries performed during the study period (2018–2023). Table 5. presents the type of surgery with SSI occurrence results. The difference between observed and predictable gastrointestinal infections was 12.1%, indicating a significantly higher incidence of SSIs (*P* < .05). Among GIs, the most frequently encountered surgeries were colorectal ones, including colostomy, colectomy, and hemicolectomy.

**Table 5.** Type of surgery with SSI occurrence results

Type of surgery	Total observed number of infections	Observed percentage of infections (%)	Predictable^‡^ percentage of infections (%)	Delta (difference in observed and predictable percentage of infections)	*P*-value
○**Gastrointestinal (general surgery)**	26	32.1	20.0	12.1%*	
○**Gynecological**	6	7.4	15.4	−8.0%*	
○**Orthopedic**	36	44.4	37.4	7.0%	
○**ENT**	7	8.6	8.2	0.4%	
○**Neurosurgery**	3	3.7	2.7	1.0%	
○**Vascular and heart**	3	3.7	6.1	−2.1%	
○**Plastic surgery**	0	0.0	1.7	−1.7%	
○**Urology**	0	0.0	8.6	−8.6%	
					**0.006**

^‡^Predictable based on surgeries distribution data collected from infection control during the period covered (2018–2023).

While GIs accounted for the highest number of SSIs, we evaluated the appropriateness of the preoperative antibiotic regimen used. Only 11 cases (42.3%) received appropriate antibiotics and doses per LAUMC-RH protocols, with cefazolin missing in 14 cases (53.8%) and metronidazole in 11 cases (42.3%). However, 25 cases (96.2%) received the correct antibiotic dose. Over the five years, the most frequent microorganisms were 12 *Escherichia coli* infections (83.3% ESBL-producing, 25% AmpC producers), 12 *Enterococcus faecalis* infections (58.3% ampicillin-sensitive, 100% vancomycin-sensitive), and 11 *Staphylococcus coagulase-negative* infections (45.5% methicillin-resistant).

### Secondary endpoints

Microbiological analysis was performed to identify wound site microorganisms and their susceptibility pattern, showing that 48% were gram-negative and 52% were gram-positive bacteria. Among the gram-negative organisms, *Escherichia coli* was the most common (26%), followed by *Enterobacter cloacae* (16%), *Klebsiella pneumoniae* (15% (10)), and *Pseudomonas aeruginosa* (13%). Notably, 83.3% of *E. coli* isolates were ESBL producers, with 16.7% also carrying the AmpC gene. Among *Klebsiella pneumoniae*, 50% were ESBL producers, 40% carried the AmpC gene, and 10% (1/10) were carbapenem-resistant *Klebsiella pneumoniae*. *Pseudomonas aeruginosa* demonstrated susceptibility rates of 77.8% to cefepime, 66.7% to amikacin, 62.5% to piperacillin/tazobactam, and 55.6% to aztreonam and imipenem. For gram-positive bacteria, the majority were *coagulase-negative staphylococci* (27%), followed by *Enterococcus faecalis* (24%) and *Staphylococcus aureus* (8%). All *S. aureus* isolates demonstrated 100.0% susceptibility to oxacillin, indicating that they were methicillin-sensitive *S. aureus*. Of the *coagulase-negative staphylococci*, 37.5% were oxacillin-sensitive, indicating 62.5% were methicillin-resistant. Approximately 28.6% of *E. faecalis* isolates were ampicillin-resistant, with no cases of vancomycin-resistant enterococci detected. When organisms were examined by type of surgery, 20% of orthopedic SSIs were caused by *Enterococcus faecalis*, making it the most common pathogen in this group. In ENT surgeries, 28% of infections were attributed to *Klebsiella pneumoniae*, *Pseudomonas aeruginosa*, and *Streptococcus oralis*. For gynecological procedures, *Escherichia coli* accounted for 33% of SSIs and was the most frequently isolated microorganism.

## Discussion

Surgical site infections remain a major challenge in the care of surgical patients, contributing to extended hospital stays, higher healthcare costs, and negative patient outcomes.^17^ This study aimed to identify the risk factors associated with SSIs, analyze the microbiological profiles of the pathogens involved, and determine their susceptibility patterns.

In our study, the rate of SSI over the last five years was notably low at 0.005%. Several risk factors were identified as potential contributors to the development of SSIs. Notably, elderly patients (≥65 yr) and those with hypoalbuminemia (<3.5 g/dL) were found to have a significantly higher risk of SSIs. These findings are consistent with previous studies highlighting that advanced age resulted in an SSI that was associated with a greater than 3–5-fold increase in mortality and greater than 10–12 days of additional hospitalization compared to the general surgical population.^18,19^ A previous study also showed that hypoalbuminemia (<3.5 g/dL) was associated with 2-fold risk of SSI in patients undergoing thoracolumbar spinal and sacral surgery; reflecting that malnutrition is associated with an increased risk of developing SSI after spinal surgery.^20^

The notably low SSI rate observed in our cohort should be interpreted in the context of our institutional surveillance and reporting processes. Throughout the study period, SSI cases were systematically reported to the infection control team on a monthly basis. However, SSI identification relied primarily on cases diagnosed during inpatient admissions. Our study did not capture outpatient postoperative follow-up or infections managed in external wound clinics, which may have resulted in under-ascertainment of SSIs.

Furthermore, our analysis suggests that antibiotic redosing may play a protective role against SSIs whenever necessary. This underscores the importance of adhering to antimicrobial prophylaxis guidelines, particularly whenever the surgery exceeds two half-lives of the antibiotics of choice.

Compliance with surgical site infection prevention bundle checklists remains an issue. While this study could not conclude a direct link between checklist adherence and SSI reduction, a systematic review indicated that checklist use can reduce SSIs from 3.2% to 1.02%.^21^ The WHO Surgical Safety Checklist has been widely adopted to mitigate surgical adverse events.

Targeted interventions are crucial, especially for surgeries with high SSI rates. GIs had significantly higher infection rates from 2018 to 2023. This finding is consistent with a previous study which highlighted that the highest prevalence of SSI was associated mostly with abdominal surgeries with a 35.6% rate.^22^ Notably, an assessment of the preoperative antibiotic regimen revealed that only 42.3% of cases utilized the appropriate antibiotics and doses according to our protocol, with over half of the cases had missing antibiotic coverage. This discrepancy highlights the need for better adherence to antibiotic protocols to reduce SSIs in GIs. Additionally, *E. coli* and *E. faecalis* are the most detected gram-negative bacilli and gram-positive cocci, respectively, at abdominal wound sites. This finding aligns with the study by Mohan et al., which reported a 100.0% proportion of *E. faecalis* and 70.0% of *E. coli* at abdominal sites.

Microbiological analysis was conducted to identify the microorganisms present at the wound sites in cases arm. 83.3% of E. *coli* and 50.0% of *K. pneumoniae* isolates were identified as ESBL producers, and 10.0% of *K. pneumonia* were KPC. These findings are consistent with a previous study conducted in Lebanon which showed that ESBL producers were identified as the most common causative agent of wound infections, and both *E. coli* and *K. pneumoniae* are among the most frequent ESBL-producing Enterobacteriaceae in these infections.^23^

Our study has several limitations. First, it is a single-centered study, limiting the generalizability of our findings to other settings. Secondly, the retrospective nature of the study may introduce biases related to the accuracy and completeness of the recorded data. Additionally, the study did not assess outpatient cases from wound clinics and focused solely on patients admitted with SSI. Finally, the study excluded the pediatric population, restricting the applicability of the findings to adult patients only. In terms of strengths, by matching cases and controls on certain variables, by effectively controlling for confounding factors, the internal validity of the study findings was enhanced. Additionally, both patient-related and procedural factors were evaluated, hence, providing a nuanced understanding of SSIs.

## Conclusion

In conclusion, SSIs remain a major challenge, leading to extended hospital stays and increased healthcare costs. This study identified that elderly patients (≥65 yr) and those with hypoalbuminemia (<3.5 g/dL) have a significantly higher risk of SSIs. Antibiotic redosing was found to be potentially protective against SSIs, underscoring the importance of following antimicrobial prophylaxis guidelines. Compliance with the SSI prevention bundle checklist was identified as an issue. Therefore, it is necessary to develop and implement targeted interventions to ensure healthcare team members in the hospital setting adhere to the surgical site infection prevention bundle checklist.

## Data Availability

The data are available from the corresponding author on reasonable request.
